# Animal models of chronic traumatic encephalopathy

**DOI:** 10.2217/cnc-2016-0031

**Published:** 2017-05-19

**Authors:** Kelly M McAteer, Renee J Turner, Frances Corrigan

**Affiliations:** 1 Discipline of Anatomy & Pathology, Adelaide Medical School, University of Adelaide, Adelaide, Australia

**Keywords:** chronic traumatic encephalopathy, controlled cortical impact, fluid percussion, tau, traumatic brain injury, weight drop

## Abstract

Repeated head impacts have been suggested to be associated with the development of the neurodegenerative disorder, chronic traumatic encephalopathy (CTE). CTE is characterized by the accumulation of hyperphosphorylated tau within the brain, with accompanying cognitive and behavioral deficits. How a history of repeated head impacts can lead to the later development of CTE is not yet known, and as such appropriate animal models are required. Over the last decade a number of rodent models of repeated mild traumatic brain injury have been developed that are broadly based on traditional traumatic brain injury models, in controlled cortical impact, fluid percussion and weight drop models, with adaptations to allow for better modeling of the mechanical forces associated with concussion.

Concussion is defined as a subset of traumatic brain injury (TBI) that is induced by biomechanical forces and results in a complex series of pathophysiological processes affecting the brain [1]. It is typically caused by a direct blow to the head, face, neck or other part of the body with an impulsive force transmitted to the head, resulting in the rapid onset of acute impairment of neurological function that resolves spontaneously [2]. These clinical symptoms may or may not involve loss of consciousness and can also include headache, changes in behavior, amnesia and insomnia [3].

Recent studies have highlighted a steady increase in sport related concussion hospitalizations, with an average annual increase of 5.4% in hospitalization rates in Victoria over a 9-year period [4]. Football codes, including rugby, Australian football and soccer accounted for 36% of concussion related hospitalizations between 2002 and 2011 [4]. An estimated 1.6–3.8 million sport related concussions occur in the USA each year, however this number is believed to be severely under reported, with up to 50% of concussions going unreported [5].

High levels of public concern regarding concussion, especially within professional sporting circles, have sparked an increased research presence within the past few years. This is due to the recent link associating participation in contact sports, exposure to repeated events of concussion and the later development of dementia-like symptoms in the years following the initial event [6].

## Link between repeated concussion & later neurodegeneration

Contact sports have long been linked to the later emergence of disturbances in cognitive function [7], with the first such instance noted in boxers in a study from 1928 describing athletes that appeared ‘punch-drunk’ in nature following repeated blows to the head [8]. The condition was termed *dementia pugilistica* in 1937 [9], and was considered neuropathologically distinct from other neurodegenerative diseases in a study from 1973 [10]. The link between repeated concussion and later neurodegeneration then returned to the spotlight with reports of distinct neuropathology within former professional American football players (NFL), as well as others exposed to repetitive concussion including wrestlers, soccer players, rugby players and those in the military [11–14]. A key paper by Omalu *et al.* reported the presence of diffuse Aβ plaques, neurofibrillary tangles (NFTs) and tau-positive neuritic threads in neocortical areas in a former NFL athlete who had a history of cognitive impairment, mood disorder and parkinsonian symptoms before death [11]. The pattern of tau deposition is distinct from other neurodegenerative diseases, with NFTs, thorned astrocytes and dystrophic neurites aggregating in the superficial cortical layers of the brain, particularly at the base of the sulci and surrounding blood vessels [15], with this presentation now known as chronic traumatic encephalopathy (CTE). Additional neuropathological features of CTE include deposits of phosphorylated TDP-43 as reactive neuronal cytoplasmic inclusions, persistent neuroinflammation, evidence of axonal injury particularly within the deep cortex and subcortical white matter, as well as loss of white matter, most evident in the corpus callosum [16]. This is accompanied by gross atrophy, most pronounced in the frontal, temporal and medial lobes [6]. CTE has been classified into four distinct disease stages that result in an increase in both the severity of clinical symptoms of patients and the associated neuropathology (Table 1). It should be noted that the diagnosis of CTE as its own distinct neuropathology is still under scrutiny and the incidence of what is believed to be pure CTE diagnoses is still unknown.

**Table 1.** **Proposed progression of chronic traumatic encephalopathy stages.**

**Stage**	**Clinical Features**	**Gross pathological changes**	**Pattern of tau deposition**	**TDP-43 immunoreactivity**	**Axonal injury**
I	Loss of attention & concentration, increased aggression	None	Focal epicenters of perivascular pTau in the sulcal depths limited to the superior & dorsolateral frontal cortices	None	Minimal

II	Depression, mood swings, short-term memory loss, loss of attention & concentration, aggression	No cerebral atrophy, mild enlargement of ventricles	pTau pathology in multiple discrete foci of the cortex. Some small NFTs present in hypothalamus, hippocampus, thalamus and SN	Some TDP-43 immunoreactivity	Minimal

III	Memory loss, executive dysfunction, explosivity, loss of attention & concentration, depression, mood swings, aggression	Mild cerebral atrophy with dilation of ventricles, septal abnormalities, atrophy of the mammillary bodies & thalamus, thinning of the corpus callosum	NFTs widespread throughout the cortex, hippocampus and amygdala. NFTs also observed in olfactory bulbs, hypothalamus, mammillary bodies and SN	TDP-43 reactive neurites observed in cerebral cortex, medial temporal lobe & brainstem	Axonal loss & distorted axonal profiles observed in subcortical white matter (frontal & temporal cortices)

IV	Executive dysfunction, memory loss, severe memory loss & dementia, profound loss of attention & concentration, aphasia, explosivity, aggression, paranoia, depression, visuospatial difficulties, suicidal tendencies	Atrophy of the cerebral cortex & white matter, medial temporal lobe, thalamus, hypothalamus & mammillary bodies. Ventricular enlargement, cavum septum pellucidum	Severe pTau abnormalities widespread throughout cerebellum, diencephalon, basal ganglia, brainstem & spinal cord	Severe TDP-43 immunoreactivity in cerebral cortex, medial temporal lobe, diencephalon, basal ganglia & brainstem	Marked axonal loss in subcortical white matter tracts with distorted axonal profiles

Adapted with permission from [14].

NFT: Neurofibrillary tangle; SN: Substantia nigra.

Although diagnosed postmortem, CTE has been linked to two types of clinical presentations, with manifestation of symptoms years, sometimes decades, after the repetitive concussions were sustained [17]. The first type of presentation manifests earlier in life at approximately 40 years of age and involves changes in mood, such patients are usually more aggressive, impulsive, physically and verbally violent and depressed [17]. The second type of presentation manifests at a much older age than the first, at approximately 60 years of age and involves changes in cognition, showing impairments in episodic memory with patients in this category more likely to develop dementia than in the first [17]. Regardless of the type of initial presentation patients will progressively develop symptoms from both groups. In some cases, patients may also develop Parkinsonian-like symptoms including tremors [18].

The underlying mechanisms for how concussion, in particular repeated exposure to concussion, may predispose to later neurodegeneration with its associated accumulation of pathological proteins, particularly phosphorylated tau, is still not understood. The number of injuries required, the intensity of injuries, the impact of concussive versus subconcussive injuries and the effect of other confounding factors such as pre-existing medical conditions and substance abuse on the development of CTE are not yet known. Furthermore, biomarkers for the disease to identify at risk individuals have also not yet been developed. Thus, in order to facilitate a better understanding of disease progression, animal models of repetitive TBI are required that replicate key aspects of the clinical situation.

## Suggested requirements for an appropriate animal model of repetitive concussion

A number of criteria have been proposed to allow animal models of concussion to be reflective of the type of injuries seen clinically. Optimally it has been suggested that the head should be struck directly and the impact should occur with high velocity and rapid acceleration of the head, both rotational and angular [19,20]. Striking the head directly causes higher accelerations of shorter durations [21], with biomechanics studies, principally in NFL footballers suggesting that angular acceleration of the head in the coronal plane has the strongest association with concussion due to generation of the greatest amount of shear force. In regard to the force required, the degree of linear acceleration required for a concussive injury is reported to be close to 100 G [22–25], and is similarly regardless of whether it is reported in helmeted NFL players or in unhelmeted athletes [22–23,26], like Australian Rules Football players [25]. In addition, the range for angular acceleration has been reported as 5022–7912 rad/s^2^ [23–24,27–28], providing a guideline for the types of forces that should be generated in animal models.

It should be acknowledged that it is difficult to translate rotational acceleration forces reported in humans to animal models, given the differences in brain size. Inertial effect is dependent on brain mass, and this determines the degree of tissue deformation [29]. As such the same forces applied to a smaller brain produce lower strains and less injury [30,31]. Indeed in the rapid nonimpact inertial head injury models that have been developed to date, injury parameters have been scaled, with the rotational acceleration increased 500% for a 140 g baboon brain [32] and 630% for a 90 g pig brain [33] to induce the same tissue strains that cause axonal injury in humans. This makes it difficult to produce equivalent rotational acceleration in small animals such as rodents with reports that to achieve equivalent tissue strains in the 2 g rat brain accelerations >5000% of that in human TBI would be required to produce similar tissue strains [34].

Instead animal models can aim to replicate the clinical features of concussion, with acute symptoms of concussion clinically encompassing physical signs such as loss of consciousness, somatic symptoms including headache and vertigo, behavioral changes encompassing cognitive impairment, irritability and sleep disturbance [1]. Evidently many of these measures are difficult to measure in animal models where animals are typically anesthetized, although some newer models are moving toward injury in awake animals [35]. Loss of consciousness is typically seen as an increase in time spent to regain righting reflex, with differences reported between sham animals and injured controls even with anesthesia [36–43]. Further advances in the field could be shifting toward more acute behavioral testing (on the same day) and monitoring of sleep patterns to allow a more complete understanding of the acute effects of our current concussive models. In addition, it has been suggested that given that by definition concussion does not cause structural abnormalities on standard neuroimaging [1], similarly animal models should be mild enough so that they do not cause more severe signs of injury such as contusions, edema or hemorrhage [44,45].

In regard to modeling repeated concussive events, the number, severity and timing between injuries also needs to be considered. It has been suggested that the optimal model would involve impacts beginning in adolescence and continuing sporadically over a long period of time [44], a pattern that has not yet been utilized. Currently animal models typically utilize short spacings between impacts (24 h–7 days) [35,37,46], in line with evidence of a window of vulnerability following a single concussion, where a subsequent concussion can have greater long-term effects [47]. However it is not known whether in addition multiple concussive and/or subconcussive impacts spaced at greater intervals could also have long-term consequences in our animal models and whether this may now be more relevant clinically given the advancements in requiring rest periods before returning to play. It has been suggested that cumulative exposure to trauma, as in the number of years of engaging in contact sport, rather than the number of concussions, is linked to the severity of tau phosphorylation [48], suggesting a key role of subconcussive hits and that CTE is primarily linked to a long history of repeated head impacts rather than a small number of concussive events in a short space of time. Investigation of the number of head impact in collegiate level American football found that players received up to 1444 head impacts in one season with an average of 6.3 impacts per practice and 14.3 impacts per game sustained per player [49]. Evidently the vast majority of these impacts are subconcussive, with reports of concussion rates ranging from 1.86 [50] to 4.46 [51] per 1000 athlete exposures. This suggests an area that needs to be further explored in our current models, with a number of studies now trying to incorporate a larger number of less severe impacts to attempt to model this clinical situation.

A further complicating factor is the difference in life span between rodents and humans and how to accurately replicate the time-course of the disease. There is typically a gap between a history of repeat injury and onset of symptoms, with behavioral symptoms reported at around 40 years of age and cognitive symptoms at 60, although noticeable tau pathology has been reported in much younger athletes [14,17]. Compared with an average human life span of 80 years, laboratory rodents live about 2–3.5 years (average 3 years) [52]. In most animal models, injury is induced in young adulthood (10–12 weeks) and at most animals are followed up to 1 year post injury (∼15–16 months) [52]. However this only equates to early middle age in humans, with the need for further studies examining up to 18–24 months post injury, to allow a complete examination of the evolution of the neurological changes induced by repeated head impacts. Furthermore modeling of spacing between injuries is complicated by the differing lifespan. Direct calculation comparing the length of adulthood in rodents and humans, leads to the calculation that 11.8 rodent days are roughly equivalent to one human year [53]. However, if this is used as the basis to determine how far apart head impacts should occur, it ignores the evolution of secondary injury factors following an insult that play a role in the effects of a subsequent insult. For instance Shultz *et al.* utilized a 5 days gap between their insults, as this allowed for complete resolution of the inflammatory response between impacts [41]. This was equated to an event that took roughly two weeks in humans, rather than the approximately 6 months which would be calculated by chronological age of the rodent. It is evident that this is a key difficulty that needs to be taken into account, and acknowledged as a limitation of the current animal models.

Regardless of their limitations animal models of concussion are required to allow us to develop an insight into the long-term effects of repeated head impacts. Animal models of repeated concussion should also be highly reflective of the current descriptions of CTE, leading to progressive cognitive deficits, mood changes and the gradual appearance of key neuropathological features such as NFTs [14,54–55], changes in white matter integrity [56] and sustained neuroinflammation [49]. Many studies have attempted to scale down current models of severe TBI, such as the controlled cortical impact (CCI) and fluid percussion (FP) injuries and weight drop models, with modifications to replicate key features seen clinically.

## Current animal models of repeated concussion

### Controlled cortical impact

A popular model of injury used currently in the development of repetitive mild TBI (mTBI) animal models is a modified version of the CCI model of TBI. Classically, CCI involves the use of a rigid impactor to deliver mechanical energy to the dura of the brain, exposed via a craniotomy to an animal restrained in a stereotaxic device to produce a focal contusive injury [57]. To replicate a concussive injury the model has typically been adapted to negate the need for a craniotomy, with use of rubber or silicone tips to allow impact to the skull directly [36,43,58–60] or to a form fitting steel cap [35,61], without generating an overt focal necrotic lesion. This is important as secondary impacts are delivered in the same location as the first, which would be confounded by the presence of a contusive injury. Indeed, the only report of significant cerebral hemorrhage and extensive cortical tissue loss in a CCI model of rmTBI was when direct impact to the dura rather than the skull occurred [62], suggesting that a more replicative injury is produced when a craniotomy is avoided. Similarly it appears that larger tip sizes in mature rats (6–10 mm) [36,59–61] produce less focal structural damage and thus may be more appropriate for reproducing concussive insults, with only smaller tips sizes associated with development of areas of hemorrhage [43,62], although this was prevented when strike depth was decreased to 1 mm. [58].

A potential criticism of these models is that although the head is struck directly, the model is less able to generate either rotational or linear acceleration forces due to the typical placement of the head within a stereotaxic device, and thus cannot model the mechanical forces that typically induce concussions clinically [44]. To assist in this modifications have been utilized within some studies, including replacement of the stereotaxic device with a molded, gel filled base [36] or placement of animals within a plastic restraint cone on a foam bed [35] to allow more movement of the head, with further characterization needed to analyze the specific types of forces generated.

Studies employing modifications of the CCI model to generate repeated concussion currently use a range of impact parameters, with acceleration ranging from 3.5 to 6 m/s and dwell time from 31.5 to 500 ms. Notably many studies do not provide objective measurements of injury severity, such as the presence of an apneic period or loss of righting reflex (LORR) [35,59,60,62], making it difficult to compare studies utilizing different injury parameters or to assess whether the impacts produced are likely to be concussive or subconcussive. Similarly injury schedules vary (see Table 2), with some employing a small number of injuries (2–5) with interinjury intervals ranging from 24 to 72 h [36,42,43,59,60,62,63], while three studies explored the effects of larger number of injuries: 30 at 24 h intervals [36], six impacts 2 h apart for 7 days [35] or 24–32 over 3–4 months [64]. Indeed it is evident that the interinjury interval can have significant effects, with Winston *et al.* finding that there was a greater effect on synaptic loss with an interinjury interval of 7 days rather than 24 h with a large number of injuries [36]. This appears counter-intuitive given the known window of vulnerability whereby a second injury has been shown to have more lasting impacts [47], but perhaps indicates some adaptation when a large number of injuries are sustained within a short space of time, which is not seen with the longer interval. Further investigation will be needed to see the effect of a large number of injuries at greater intervals on other aspects seen in CTE, such as behavioral changes and tau phosphorylation.

**Table 2.** **Summary of studies utilizing modified versions of the controlled cortical impact model to produce repeated head impacts.**

**No. of injuries**	**Injury interval**	**Impact parameters**	**LOC**	**Tau pathology**	**Axonal injury**	**Neuroinflammation**	**Effect on cognition**	**Anxiety and depressive like behavior**	**Motor changes**	**Ref.**	
30 20	24 h	5 mm depth, 2.35 m/s, 31.5 ms dwell time, 10 mm Teflon tip	Yes	No increase in pTau in 18 month 3xTgAd mice at 1 month post injury	None	Increased microglial proliferation and activation at 30 days, persisting to 1 year post injury	None	Increased anxiety as seen on EPM at 1 year, but not 6 months post injury	Motor deficits at 1 year post injury, as seen as reduced time to fall from rotarod	[36]	

3	24 h	3 mm depth, 4 m/s, 200 ms dwell time, 9 mm, rubber tip	–	Increased pTau in the ipsilateral corpus callosum, cortex, hippocampus, septal nucleus and amygdala	–	Significant astrogliosis 6 months post injury in cortex, hippocampus and corpus callosum	Impaired MWM performance at 2 months post injury. Impaired cued and contextual memory on fear conditioning test at 6 months post injury	None	None	[59]	

2	3 days	0.5 mm depth, 6 m/s, 200 ms dwell time, 4 mm tip	–	–	–	Increased astrocytic and microglial immunoreactivity at impact site at 14 days post injury	Learning deficits in accelerated, but not standard MWM at 30 days post injury	–	None	[65]	

2	24 h	3.3 mm depth, 5 m/s, 100 ms dwell time, 9 mm rubber tip	–	–	Mild axonal injury within the ipsilateral cortex and external capsule at 7 days post injury	Increased IBA1 immunoreactivity in regions corresponding to positive sliver staining. Mainly resolved by 49 days except in corpus callosum	Significant impairments in MWM at 3–6 days post injury, with improvement by 7 weeks post injury	–	–	[60]	

5	24 or 48 h	2 mm depth, 3.5 m/s, 500 ms dwell time, 5 mm silicon tip	Yes	Not seen	Mild axonal injury in white matter tracts within the cerebellum, and scattered throughout the brainstem, more pronounced with 24 h injury interval	Astrogliosis and microglial activation observed in 24-h interval animals at 24 h post injury in the entorhinal cortex and cerebellum	–	–	–	[43]	

42	2 h	1 cm depth, 5 m/s, 100 ms dwell time, 6 mm rubber tip	–	Increased pTau to 6 months post injury in cortex and amygdala. Evidence of increased pTau peaking at 1 month and residing by 6 months in hippocampus	–	Evidence of astrogliosis and increased microglial reactivity within the cortex, amygdala and hippocampus at 7 days post injury, that subsided by 1 month and then re-emerged at 6 months post injury	Persistent deficits on MWM evident from 1 week to 6 months post injury	Decreased anxiety as assessed on EPM at 1 and 6 months post injury More time spent immobile on FST at 1 month post injury	Vestibulomotor disturbance on wire grip test acutely post injury	[35,61]	

2	24 h	8 mm depth, 36 psi, 5 mm tip	Yes	–	Increased axonal injury at white-gray matter interface (24 h post injury)	Increased GFAP immunoreactivity in white-gray matter interface	Cognitive deficits on NOR, but no different to single injury animals	–	–	[63]	

5	24 or 48 h	2 mm depth, 3.5 m/s, 500 ms dwell time, 5 mm silicon tip	Yes	No elevations in pTau	Chronic axonal degeneration in the cerebellum, optic tracts	Increased microglial reactivity in brainstem and cerebellum, but not hippocampus at 70 days post injury	Persistent cognitive deficits on NOR to 70 days post injury	–	Persistent motor deficits to 70 days on beam walking tasks	[66]	

24 or 32	2 impacts weekly for 3–4 months	1.0 mm depth, 5 m/s, 200 ms dwell, 5 mm tip	Yes	Increased pTau within the cortex, but not white matter 3 months post injury	Chronic axonal injury within the corpus callosum	Activation of microglia and astrocytes within corpus callosum				[64]	

5	48 h	1.0 mm depth, 5 m/s, 200 ms dwell, 5 mm tip	Yes	Increased pTau within the cortex and hippocampus at 3 weeks post injury	–	Increased GFAP and CD45 immunoreactivity within the cortex and hippocampus	–	–	–		

5	48 h	1.0 mm depth, 5 m/s, 200 ms dwell, 5 mm tip	Yes	–	Evidence of axonal injury within corpus callosum and brainstem acutely	Increased GFAP and IBA1 immunoreactivity within the cortex and hippocampus	Cognitive deficits on MWM a fortnight following injury	–	Motor deficits on the rotarod acutely, resolving within a month	[58]	

–: Not reported for a study; EPM: Elevated plus maze; FST: Forced swim test; LOC: Loss of consciousness; MWM: Morris water maze; NOR: Novel object recognition.

Nonetheless many of these models do replicate some features associated with the sequelae of repeated concussion, and importantly many look at the long-term effects (up to 1 year post injury), which is important when attempting to replicate CTE such as neuropathology. Unsurprisingly all studies reported persistent neuroinflammation, as seen by increased astrocytic and microglial reactivity following repeat injury [36,59–61], although evidence of enhanced tau phosphorylation, the key diagnostic feature of CTE was not consistently reported. Luo *et al.* saw increased pTau immunoreactivity in regions including the hippocampus and cortex at 6 months following three injuries spaced 24 h apart, while Petraglia *et al.* similarly reported enhanced tau phosphorylation at 6 months post injury in the cortex and amygdala, whereas hippocampal pTau had subsided at this point, despite a vastly different injury schedule (42 impacts in 5 days). In contrast Winston *et al.* found that delivering 20 impacts over a 4 week period (5 daily impacts a week) to 18 month 3xTgAd mice did not cause an increase in levels of pTau at 24 h or 1 month following injury. Of note repeated injury is seen more commonly in younger populations and age at impact may affect the likelihood of increasing tau phosphorylation. Indeed Ojo *et al.* utilizing transgenic 12 weeks old htau mice found significantly increased levels of phosphorylated and aggregated tau at 3 months post injury within the cortex when mice were exposed to highly repetitive mTBI (24 or 32 impacts within 4 months) [64].

Other key aspects of CTE are the development of behavioral symptoms including increased anxiety, depression and cognitive deficits [14]. The majority of papers reported cognitive deficits, although these appeared shortly after injury (in the first week), with some reporting improvement at later time-points [60] or persistence to 6 months post injury [59,61]. None saw the emergence of cognitive deficits over time [59–61], which would represent a more consistent pattern to what is seen clinically [17] and may indicate that the current CCI models are too severe, but could also relate to the difficulty in detecting subtle cognitive deficits in rodents. Notably Winston *et al.* who did not find evidence of cognitive deficits on the Morris water maze (MWM), did see an emergence of anxiety-like behavior at 1 year post injury that was not evident at 6 months, suggesting a progressive, rather than static, disease course [36]. As such, it is evident that there are a number of models based on modification of the CCI device that can replicate aspects seen clinically following rmTBI, although there are also limitations due to the difficulty in generating the same mechanical forces seen clinically.

### FP injury

The lateral FP (LFP) model is the most extensively used and characterized model of experimental TBI and is easily adapted to produce milder injuries by decreasing the force of the fluid pulse [67]; however, there are relatively few studies utilizing it to investigate the effects of repeated injury [40,41,46,68,69]. Injury is induced with FP by performing a craniotomy and applying a fluid pressure pulse to the intact dura, caused by the striking of a pendulum against a piston attached to a reservoir of fluid, producing displacement and deformation of neural tissue [70]. As such, it does not reproduce the linear and rotational forces that generate concussive injuries clinically.

Previous literature has suggested that mild to moderate injury can be administered between (0.9–2.1 atm) [71], and indeed reports using LFP to induce repeated mTBI herein use pressures that fall within the smaller end of this scale (1.0–1.5 atm; Table 3). One difficulty with use of the model for repeat injuries is the necessity for a craniotomy to be performed to administer the injury. This increases the risk of other factors such as wound infection and the animals removing the screw/cement complexes that are necessary to induce injury [69]. Furthermore it limits the number of injuries that are able to be delivered with five injuries the highest reported [41], unlike the modified CCI models and weight drop models. In addition repeated LFP appears to cause significant cortical damage, when impacts are spaced closely together (within 24 h) or when more than three impacts are given spaced 5 days apart [40,41,46], a feature not suggestive of the type of pathology seen in CTE, which is a progressive neurodegenerative disease.

**Table 3.** **Summary of studies utilizing the lateral fluid percussion injury model to produce repeated head impacts.**

**Number of injuries**	**Injury interval**	**Impact parameters**	**Tau pathology**	**Axonal injury**	**Neuroinflammation**	**Effect on cognition**	**Anxiety and depressive-like behavior**	**Motor changes**	**Ref.**
3 or 5	5 days	1.0–1.5 atm, 5 mm craniotomy	–	–	Increased activated microglia within the cortex at 24 h and 8 weeks in 5 rmTBI animals. 3 rmTBI animals only showed increased activation at 24 h	Impaired performance in MWM both within the first week following injury and at 8 weeks post injury in 3 and 5 rmTBI groups	Increased depression as measured on FST and anxiety on EPM in 5 rmTBI animals at 8 weeks post injury	None (beam task)	[41]

2 or 3	5 days	1.0–1.5 atm, 5 mm craniotomy	Increased cortical pTau at 24 h and 1 week following 2 mTBI, returning toward baseline at 1 month	Decreased corpus callosum size, and decreased integrity of its white matter tracts as seen with DTI	–	Impaired performance on MWM at 3 months post injury (3 mTBI)	Increased depression as measured on FST	Increased slips and falls on beam task at 3 months post injury (3 mTBI)	[46]

3	5 days	1.0–1.5 atm, 5 mm craniotomy	Increased pTau in the ipsilateral corpus callosum, cortex, hippocampus, septal nucleus and amygdala	Decreased integrity of white matter in corpus callosum as seen with DTI	Increased GFAP and CD68 immunoreactivity at 3 months post injury	Impaired performance on MWM at 3 months post injury	–	Increased slips and falls on beam task at 3 months post injury	[40]

2 or 3	10–14 days	1 atm, 3 mm craniotomy	–	–	–	Worsening MWM performance acutely with increasing number of concussions	–	None	[69]

2	24 h, 5 days or 15 days	1.14 atm, 5 mm craniotomy	–	–	Increased cortical GFAP and IBA1 immunoreactivity at 21 days post injury (24 h and 5 days interinjury interval	–	–	Impaired motor function acutely with rmTBI at 24 h interval	[68]

–: Not reported for a study; DTI: Diffusion tract imaging; EPM: Elevated plus maze; FST: Forced swim test; mTBI: Mild traumatic brain injury; MWM: Morris water maze; rmTBI: Repeated mild traumatic brain injury.

However, some features reported to be related to the long-term effects of repeated concussion, with persistent neuroinflammation to 3 months post injury (the latest time point studied) [40–41,68], evidence of white matter damage [40,46] and increased tau phosphorylation within the cortex [46]. It should be noted that with two injuries spaced 5 days apart increased tau phosphorylation was more prominent acutely after injury (at 24 h and 1 week) returning toward sham level by 1 month post injury, with an increase to three repeat injuries associated with increased tau phosphorylation to 3 months post injury [46]. Like the modified repeat CCI models, multiple LFP injuries are associated with cognitive deficits both acutely [41,69] and chronically [40,41,46,69], although this model has been associated with more significant motor impairments most likely related to the degree of cortical injury [40,46,68].

### Closed head weight drop

Closed head injury models involve the application of force directly onto the intact skull, which causes movement of the unrestricted head, including lateral and rotational forces, as seen in concussive insults. This produces a diffuse injury, with no reports of cortical contusions of hemorrhage unlike some CCI [43,62], or FPI models [40,41,46]. Typically a weight is dropped from a height, either onto the head itself, or onto a metal helmet applied to the skull to prevent the skull fracture, with head movement facilitated by placement of the animal within a foam bed [38,72,73] or by allowing the animal to free fall from the surface they were resting on (aluminum foil, Kim wipes of magnetic sheets) into a foam bed below [37,39,74]. The latter allows unrestricted movement, and hence may promote more rotational injury, which is known to cause the shear strain critical in concussive impacts, although biomechanical studies on these forces have yet to be reported. Indeed it should be noted that there are similarities between the weight drop models and modifications of the CCI, such as that utilized by Petraglia *et al.* [35,61] where the head is not restrained, with the key difference being how the force to the head is generated.

Notably all reports examined here suggest that the impacts delivered lead to increased LORR [37–39,72–74], indicative of concussive impacts and hence the more severe end of sporting injuries. Future studies could alter parameters to include subconcussive impacts to investigate the effect of combining these types of impacts. Furthermore, Briggs *et al.* who utilized the highest number of impacts (30) found that LORR decreased with subsequent impact suggesting some adaption to the impact, an important point to consider when using LORR as a measure of impact severity, especially when animals are subject to a large number of head impacts spaced close together (5 impacts/week for 6 weeks) [74].

Similar to the models discussed above, weight drop models similarly report variable neuropathological and behavioral findings associated with the long-term consequences of repeated concussion. Again this may be in part be caused by variability of the number of impacts employed, with as low as three [73] and as high as 30 [74], interinjury intervals ranging from 24 h to 5 days and the weight and hence force of impact reported as between 40–95 g in mouse studies [37–39,74] and 200–450 g in rat studies [72,73] with release of the weight typically from 1 m. As with other concussion models, increased neuroinflammation was consistently reported in studies incorporating weight drop models [37–39,73,74], up to 6 months post injury, although intriguingly both Mannix *et al.* and Kane *et al.* reported increased astrocytic, but not microglial activation at chronic time-points suggested there could be a differential response of these two immune cells [37,39]. Similar to the CCI models, increased tau phosphorylation was not a consistent feature, with reports of acutely increased pTau in some studies within areas such as the cortex, hippocampus and white matter tracts [37,72–74], while Mannix *et al.* reported normal levels of pTau at 6 months following 7 rmTBI in 9 days [39] and Xu *et al.* similarly found no changes in pTau at 10 weeks following 12 hits over 7 days [38]. Given the considerable differences within the studies that reported positive findings and those with negative tau findings (see Table 4) it is difficult to determine the key factors within injury models that allow for the development of abnormal tau phosphorylation post injury, with greater exploration of both how the mechanical forces induced and their severity and frequency influence tau phosphorylation, as well as the role of different tau phosphorylation sites may be needed in future studies. Tau can be phosphorylated at up to 85 different sites [75], so subtle changes in tau phosphorylation state can be missed depending on the antibodies utilized.

**Table 4.** **Summary of studies utilizing a weight drop injury model to produce repeated head impacts.**

**Number of injuries**	**Injury interval**	**Impact parameters**	**LOC**	**Tau pathology**	**Axonal injury**	**Neuroinflammation**	**Effect on cognition**	**Anxiety and depressive like behavior**	**Motor changes**	**Ref.**	
7 5	9 days 24 h, 1 week, 2 weeks of 1 month	54 g weight from 71 cm, animals supported on Kimwipe	Yes	No increase in pTau in cortex or hippocampus at 6 months (7 rmTBI in 9 days)	No white matter pathology detected on MRI (FA and MTR) at 6 months (7 rmTBI in 9 days)	Increased GFAP, but not IBA1 immunoreactivity at 6 months post injury (5 rmTBI in 5 days)	Impaired cognition at 1 year post injury on MWM with inter injury interval of 24 h	–	–	[39]	

4 or 12	2 h– 3 days	40 g weight from 1 m, animals supported on foam bed	Yes	No increase in tau phosphorylation in cortex or subcortex 10 weeks post injury	Axonal injury seen within corpus callosum, optic tract and cerebellum at 7 days post injury	Increased microglial activation in areas corresponding with axonal injury	–	–	–	[38]	

10	24 h	200 g weight from 1 m, animals supported on foam bed	Yes	Increased pTau in the ipsilateral corpus callosum, cortex, hippocampus, septal nucleus and amygdala	Decreased integrity of white matter in corpus callosum as seen with DTI	–	Impaired MWM performance at 14 days post injury	–	Acute motor deficits on the beam walk	[72]	

5	24 h	95 g weight, 1 m drop, animals fall through aluminum foil	Yes	Increased pTau immunoreactivity at 1 month post injury	–	Increased GFAP immunoreactivity in cortex and hippocampus at 1 month post injury, but no microglial activation	–	–	None	[37]	

3	5 days	450 g weight from 1 m, animals supported on foam bed	Yes	Increased pTau immunoreactivity in cortex under the impact site at 24 h and 3 months post injury	Increased APP immunoreactivity in cortex and thalamus	Increased astrocytic and microglial activation both acutely and chronically	Cognitive deficit on the Barnes Maze at 12 weeks, but not 6 weeks post injury	–	None (rotarod)	[73]	

30	5 daily, then a 2 day rest period	75 g or 95 g weight from 1 m, with animals supported on a platform consisting of two magnetically adjoined transparent acetate sheets	Yes^†^	Increased pTau within the corpus callosum and optic tract	Thinning of the corpus callosum	Increased microglia and astrocytes within the corpus callosum and optic tract at 53 days post injury	Impaired cognition on the Barnes Maze at 21 days post injury	Decreased coat status indicative of depressive-like behavior	Delayed development of motor deficits on the rotarod	[74]	

3	24 h	100 g weight from 70 cm	Yes	–	–	–	Cognitive deficits to 2 weeks post injury on MWM	No effect (EPM, FST)	–	[76]	

^†^Period of LOC decreased with increasing number of injuries.

–: Not reported for a study; DTI: Diffusion tract imaging; EPM: Elevated plus maze; FA: Fractional anisotropy; FST: Forced swim test; LOC: Loss of consciousness; MWM: Morris water maze; MTR: Magnetization transfer ratio; rmTBI: Repeated mild traumatic brain injury.

As discussed earlier, other key aspects of CTE are the development of behavioral symptoms including increased impulsivity, depression and cognitive deficits [14]. Like the other injury models, the weight drop model was similarly associated with cognitive deficits, both acutely within the first 2 weeks following injury [72] and persisting to 1 year post injury [39]. Mannix *et al.* did see a protective effect of increased interinjury interval with impaired performance on the MWM at 6 months following injury with a shorter gap between injuries (24 h or week), but not at longer intervals (2 weeks, 4 weeks). Furthermore, in one study a progressive cognitive deficit, suggestive of ongoing neurodegeneration was seen with increased escape latency on the Barnes Maze seen at 12 weeks, but not 6 weeks post injury [73]. Few studies looked at additional behavioral symptoms with reports of delayed motor deficits developing following a large number of impacts (30 over 6 weeks) and persistent depressive-like behavior in the same model, with further study needed, especially given the suggestion that psychological symptoms may be the first manifestation of chronic neuropathology associated with repeated head impacts [17].

### Rotational acceleration models

To date no pure rotational acceleration models (without head impact) have been developed to study the effects of repeated insults to the brain on later neurodegeneration. This is in line with the clinical literature where typically an impact to the head is received that then leads to rapid acceleration [1]. This is most closely replicated by the weight drop models where animals are free to fall from the surface they were resting on (aluminum foil, Kim wipes of magnetic sheets) into a foam bed below [37,39,74]. Nonetheless there are currently rotational acceleration models have been developed in the rat [77], rabbit [78], pig [79] and primate [80] that could be adapted to allow for milder injuries. Gutierrez *et al.* developed a rabbit model, where impact from a pneumatic cylinder was transferred to the skull surface to produce a maximal rotational velocity of 212 krads/s^2^ [78]. This led to extensive subarachnoid hemorrhage, so would need to be scaled to produce a milder injury. In rodents, Xiao-Sheng *et al.* developed a model where the head was rapidly rotated 90° in the coronal plane at a rotation reported to be 1.806 × 10^5^ rad/second^2^ [77]. It is unclear whether this would be sufficient based on the Holbourn scaling relationship to accurately represent forces seen in human TBI, given the smaller size of the rat brain [34]. Notably even at these forces, extensive subarachnoid hemorrhaging was also noted [77], suggesting that it may be difficult to accurately represent rotational forces in small animal models given the differences in size and nature of their brains. Indeed models have been developed in larger gyrencephalic brains as discussed in the next section.

## Adaptation of gyrencephalic models of mTBI to investigate repetitive insults

In addition to continuing to improve rodent models, another avenue to investigate different aspects of the effects of repetitive impacts on the later development of neurodegeneration may be to modify existing large animal models. There are structural differences between the rodent (lissencephalic) and human (gyrencephalic) brains. Importantly mechanical forces are distributed differently, with linear forces seen in lissencephalic brains concentrated parallel with the surface of the brain, compared with at the base of the sulci in gyrencephalic brains [81–83]. Computer modeling of the patterns of stress in the gyrencephalic brain [81–83] are remarkably similar to the tau deposition patterns observed in CTE, and may suggest that tau deposition occurs at areas of high mechanical stress [16] an idea that has yet to be confirmed experimentally. Indeed, to date no repeat injury models have been conducted in gyrencephalic brains, and only two model of mTBI. Browne *et al.* modified a miniature swine model of TBI where a pneumatic actuator is used to induce rotational acceleration of the head, using forces of up to 28,000 rad/s^2^ [79], higher than the reported range of 5022–7912 rad/s^2^ reported clinically [23,24,27,28], which was described as a mechanism to equate for the smaller size of the brain within the miniature swine. Nevertheless and impact within the axial plane produced a mTBI with loss of consciousness of between 10 and 35 minutes associated with mild axonal injury [79], thereby equating to a more severe clinical concussion. Older studies have also been conducted in monkeys, with acceleration of the head without impact, severity of injury depended on the direction of head movement, with a sagittal head motion producing a loss of consciousness for <15 min, without evidence of diffuse axonal injury [80]. The ability to produce concussive insults in larger animals provides a basis for further investigations utilizing large animal models to investigate aspects of repeated head impacts in the gyrencephalic brain.

## Conclusion

In recent years a desire to understand the nature of sports-related head injury has led to resurgence in interest in modeling aspects of repeated injury. This has allowed a greater appreciation of the idea of a window of vulnerability following a concussive event, whereby a subsequent concussion can have more detrimental effects and greater efforts in preventing premature return to play. However, how a prolonged history of head impacts, both concussive and subconcussive, as seen in NFL players, may increase the risk of later developing the neurodegenerative disease CTE is less clear. A number of rodent models have been generated that aim to replicate different aspects of concussive insults, with studies varying markedly in how the concussive insult is induced, the injury severity utilized, the number of insults and the interinjury variability. Unsurprisingly this had led to a variable ability to replicate key aspects of CTE, such as increased tau phosphorylation and development of cognitive and behavioral deficits. Indeed no models have been able to replicate the staged progression of tau pathology, where it begins in the superficial cortex and then spreads to other regions such as the hippocampus, or its associated features such as TDP-43 immunoreactive nuclear inclusions. This is in part due to the differences in murine and human tau, but even transgenic models have not consistently reported increased tau phosphorylation and development of NFTs. Later time points may also need to be investigated, although some reported cases of CTE have been in young players [84]. Progression of behavioral deficits has also been rarely seen in animal reports to date and area that requires further investigation. Another area of potential investigation is to determine how the gyrencephalic brain responds to mechanical insults and how this may influence tau phosphorylation and deposition and the potential later development of neurodegeneration, although there are technical limitations to the number of injuries that could be delivered in these models.

## Future perspective

Animal models of repeated mTBI will continue to evolve, and may begin to include injuries of varying severity and varying interinjury intervals to try and better approximate the clinical situation. With improving genetic technology, newer transgenic rodent models may be available to better allow modeling of tau dynamics within our rodent models. A better understanding of how a history of repeated injury may interact with lifestyle factors, such as drug addiction may also be incorporated in our models, given the vast majority of people who receive multiple head impacts do not go to develop neurodegeneration. Furthermore it is proposed that research will branch into large animal models to utilize their gyrencephalic brains to understand the differing effects of mechanical forces and how this influences tau phosphorylation.

**Executive summary**

- A history of repeated head injury is associated with the risk of later developing the neurodegenerative disease chronic encephalopathy, which involves the graded deposition of hyperphosphorylated tau, accompanied by persistent neuroinflammation and evidence of white matter damage.
- In order to understand the link between repeated head injury and later neurodegeneration, animal models have been developed to model different aspects.
- These are typically based on adaptations of traditional traumatic brain injury models in cortical impact, fluid percussion and weight drop, with modifications to suit a more concussive insult.
- Currently a wide range of different parameters are in use making comparisons between studies.
- There has been variable success in replicating key features of chronic traumatic encephalopathy such as increased tau phosphorylation and progressive behavioral deficits.
